# Walking with Neuropathic Pain: Paradoxical Shift from Burden to Support?

**DOI:** 10.1155/2015/764950

**Published:** 2015-07-28

**Authors:** David J. Kopsky, Jan M. Keppel Hesselink, Roberto Casale

**Affiliations:** ^1^Institute for Neuropathic Pain, Vespuccistraat 64-III, 1056 SN Amsterdam, Netherlands; ^2^Institute for Neuropathic Pain, Spoorlaan 2a, 3735 MV Bosch en Duin, Netherlands; ^3^Habilita, Care & Research Rehabilitation Hospitals, Via Bologna 1, 24040 Zingonia, Italy

## Abstract

Baclofen 5% cream can be used for the treatment of neuropathic pain. We describe an unusual case of a neuropathic pain patient with spinal cord injury. A 71-year-old woman with a partial spinal cord injury lesion at L4 complained of tingling, pins and needles, and burning in her legs. She scored her pain as 6 before adding baclofen 5% cream to her pain medication (pregabalin 450 mg, acetaminophen 3000 mg, and diclofenac 150 mg daily). One month later she experienced complete pain relief, though experienced increased difficulties in walking, leading to frequent falls. Her steadier walking without stumbling and falling was more important to her than pain reduction. Thus she decided to stop using baclofen. This unusual case report discusses two important issues that relate to pain medicine and rehabilitation in patients with painful spinal cord lesions: (1) the presence of wide areas of sensory loss “covered” by the presence of painful sensations and (2) pathological sensations that can be used and integrated in the body schema to create an improved spatiovisual orientation and thus mobility. Both these aspects have to be taken into account when treating pain and design rehabilitation programs.

## 1. Introduction

Neuropathic pain is usually an unsolicited symptom accompanying a disease or lesion affecting the somatosensory system such as in spinal cord injury [1]. Unfortunately, neuropathic pain can considerably affect the quality of life. The common understanding of acute pain is that it has a warning function, to protect living beings from getting more injured. Pain thus can have a useful function within the context of optimal adaptation to its environment. The effect of lacking such a protecting and adaptive mechanism can be clearly seen in congenital insensitivity to pain, leading to wounds and severe functional and structural impairments [2]. On the other hand, chronic pain states are usually regarded as a useless and troublesome side product of a disorder, such as neuropathic pain. However, might this type of useless pathology still serve a biological function in certain cases?

We will describe a partial spinal cord injury patient suffering from severe neuropathic pain. In the course of treatment with topical baclofen, we established complete pain reduction. However, the patient reported to experience more difficulties in maintaining posture and locomotion, leading to frequent stumbling and falls. We will discuss the possible cause for this unexpected effect.

## 2. Case Presentation

A 71-year-old woman with incomplete paraplegia L4 (conus cauda lesion, Asia D) due to a hernia nucleus pulposus L3-L4 underwent a laminectomy in 2010, complicated by two periods of postoperative bleeding. In April 2011, complaints of neuropathic pain in the legs worsened and subsequently an intradural cyst at L3-L4 developed, leading to a shift to the left of the cauda. Operation was not an option due to high risk of further loss of motor functions. In September 2012, the patient visited our Institute for Neuropathic Pain complaining of tingling, pins and needles, burning, and slight numbness in her legs. At the first visit she scored an 8 on the Douleur Neuropathique 4 (DN4) questionnaire, which supports that her pain was of neuropathic origin [3].

Despite pharmacological treatments with pregabalin 450 mg, acetaminophen 3000 mg, and diclofenac 150 mg at the time of the first consultation, she scored the intensity of her pain as 6 on the 11-point numerical rating scale (NRS). In the past amitriptyline and gabapentin did not have sufficient effect. She could walk 60 minutes using a walking stick. Due to paresis of the foot extensors (MRC 4/5), the patient had reduced clearance while walking. There was also reduced stability and balance due to hip extensor weakness. We decided to add baclofen 5% cream applying 2 to 3 times daily on the neuropathic pain areas, as an analgesic from a different class could provide additional pain reduction. Oral baclofen was not used due to the risk of increasing muscle weakness. One month later the patient reported that tingling, pins and needles, and burning were completely vanished after application of the baclofen cream, without experiencing any side effects such as dizziness, drowsiness, and muscle weakness. However, she also reported that her quality of life changed in a surprisingly negative direction. She experienced more difficulties in walking and stumbled around leading to frequent falls, as she did not feel pain anymore and experienced considerably increased numbness of her legs. The absence of positive neuropathic symptoms and the concomitant surge of negative neuropathic symptoms in both her legs and feet apparently prevented her from being aware of her legs and feet and thus led to an increase in locomotor difficulties. Her steadier walking without stumbling and falling was more important for her than a reduction in neuropathic pain. Thus she decided to stop using baclofen cream, although this topical treatment evidently had a robust analgesic effect. After stopping the baclofen cream her previous complaints of tingling, pins and needles, burning, and slight numbness in her legs were as before.

## 3. Discussion

This case points out three pivotal aspects that deserve separate discussion:

1. baclofen 5% cream is able to control neuropathic pain in a case with paraplegia;
2. positive neuropathic symptoms can mask underlying sensory losses and a successful treatment can disclose them;
3. elimination of painful input and the unmasking of sensory losses interfered considerably in walking ability.

## 4. Baclofen Controls Pain

Baclofen acts as an agonist on the GABA_B_ receptor, which belongs to the family of G-protein-coupled receptor and mediates slow and prolonged inhibitory transmission. Activation causes a decrease in Ca^2+^ membrane conductance and increase in K^+^, most probably due to activation of tetraethylammonium-sensitive K^+^ channels. GABA_B_ receptors are well known to be widely distributed throughout the central nervous system. In the periphery, GABA_B_ receptors are found in cutaneous layers on fine nerve endings and keratinocytes, and thus these receptors provide a new target for the topical treatment of neuropathic pain, also in patients with a lesion of the spinal cord [4].

## 5. Pain Control Unmasks Negative Sensory Symptoms

A lesion of the nervous system is always related to sensory and/or motor signs and symptoms. Both can be positive and/or negative. In case of neuropathic pain positive sensory symptoms, that is, allodynia (pain caused by normally a nonpainful stimulus), dysesthesia (abnormal sensation whether spontaneous or evoked, such as tingling, burning, pins and needles), and hyperalgesia (increased pain from a normally painful stimulus), can mask (partly) negative symptoms, for example, hypesthesia (decreased sensitivity to stimulation) or even anesthesia, manifested in numbness. In our patient complete pain control, thus complete reduction of the positive neuropathic symptoms, revealed the objective sensory nerve damage: numbness.

## 6. Shift of Functionality

Successful pain control was followed by reduction in motor functionalities, most probably not related to the antispastic effect of baclofen, as muscle strength did not wean. The functional use of pathological sensations to support the body schema and thus assist in the body equilibrium and moving around is rarely described. We speculate that complete reduction of the pathological positive symptoms and the consequent sensory “silence” from her legs made her walking unstable without a continuous visual feedback.

A possible theoretical framework to understand this phenomenon can be found in the concepts of body schema and body awareness. Body schema is defined as a central representation of spatial characteristics of the body, which include the length of body segments, their hierarchical structure, and the form of body surfaces [5]. It is suggested that our body schema does not ascend to the level of consciousness (not being aware) and is mainly needed for the unconscious dynamic architecture of our motions. Body awareness can be defined as the total of our subjective experiences of “having” a body, related to our body schema, body posture, and body position in relation to spatial motions [5]. Body awareness is the conceptualization based on the experience coming from our body and on the evaluation of the information received from different sources of entero-, proprio-, and exteroceptive inputs [5].

In this case the positive sensory inputs, even if painful, were integrated in a new pathological body schema, used for improved spatiovisual orientation and consequently walking. And thus complete control of pain, that is, the elimination of pathological inputs, did not result in better functional adaptive plasticity of the nervous system. Most probably directly reduced peripheral sensibility thereby affecting the sensory feedback did not give the patient time to adapt to her locomotion to the new situation. In slow progressive peripheral neuropathies with progressive reduction of proprioceptive feedback control, patients have the possibility to adapt their locomotion, such as reduced walking speed, prolonged double support times, and widened base widths [6]. On the other hand, a painful neuropathy also reduces the walking speed [7]. Thus pain control with still some sensory input is in this case the most preferable situation.

## 7. Neurophilosophical Framework

Our vertical spatial orientation is a key factor defining our being human, as Scheler already pointed out in 1928 [8]. Our relation to gravity is crucial for our psychophysiological integrity and defines the maintenance of upright posture, gait, and most motor activities. Three different sensory systems support our spatial orientation and behavior: the vestibular, the visual, and the somatosensory systems. Damage to the somatosensory systems, as occurs in partial spinal cord lesions, leads to abnormal perception of our body's orientation in space and can subsequently disturb posture and locomotion [9].

## 8. Reattribution

This case demonstrates how neuropathic pain is reattributed in a positive supportive sensation. Before treatment our patient sought pain relief, not realizing that in her case the pain contributed to her locomotor functions: stability and spatiovisual orientation. It was only after the positive neuropathic symptoms vanished that she noticed the important contribution of those symptoms to her locomotor functionality. Due to her experience she reattributed a positive meaning to the initially bothersome symptoms.

## 9. The Paradox: The “Meaning” of Neuropathic Pain

Buytendijk, a Dutch professor, teaching physiology, developed a concept inspired by the* Gestaltkreis* concept of Weizsäcker, a neurological fundament “to (re-)introduce the subject into physiology” [10]. Buytendijks concept of functional intentionality is that internal and external stimuli do not operate only related to their physicochemical characteristics, but especially according to their meaning for the person [11]. This concept is helpful to understand the patient's reaction. After successful therapy the patient was hampered in her spatial awareness and therefore suffered from an even greater reduction in her quality of life. Only then she recognized neuropathic pain as meaningful cues for her body schema and awareness. This case stipulates Buytendijk's concept: humans do not react to stimuli as defined by scientific physiology, but to the meaning which these stimuli have for the person [12].

## 10. Conclusion

This unusual case report underlies two important problems that link pain medicine and rehabilitation in patients with painful spinal cord lesions: (1) the presence of wide areas of sensory loss blunted by the presence of painful sensations and (2) painful sensations that can be incorporated in our body schema, to create an improved spatiovisual orientation and thus mobility. This has to be taken into account when planning both pain control and rehabilitation programs.
